# Supplementary material for: Synergistic activation by Glass and Pointed promotes neuronal identity in the Drosophila eye disc
Source: Nat Commun. 2024 Aug 17;15:7091. doi: 10.1038/s41467-024-51429-z (PMC11330500; doi:10.1038/s41467-024-51429-z)
Supplement: Supplementary file 1 — Supplementary Information [file 41467_2024_51429_MOESM1_ESM.pdf]

Supplementary Material

Synergistic activation by Glass and Pointed promotes neuronal identity in the *Drosophila* eye disc

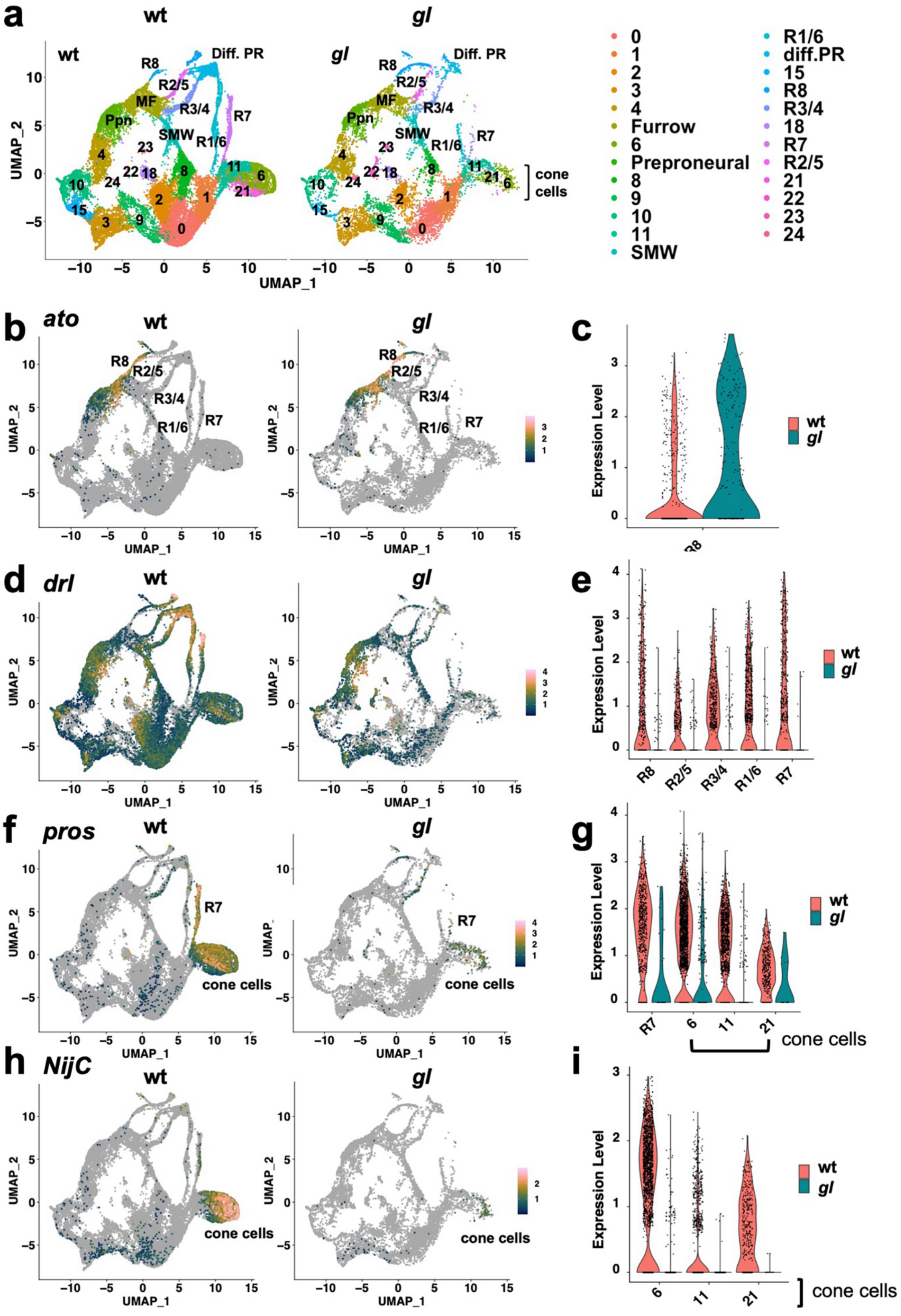

a
wt
gl
Diff. PR
R8
R2/5
R3/4
MF
Ppn
R7
SMW
R1/6
cone cells
UMAP_1
UMAP_2
0
1
2
3
4
Furrow
6
Preproneural
8
9
10
11
SMW
R1/6
diff.PR
15
R8
R3/4
18
R7
R2/5
21
22
23
24
b
ato
c
Expression Level
R8
d
drl
e
f
pros
g
h
NijC
i

Supplementary Figure 1: Loss of *gl* affects late differentiation but not R8 specification. (a) Dimension plot of integrated scRNA-Seq data of wild type and *gl* mutant with cluster annotation. Clusters 11, 21 and 6 all correspond to cone cells. (b, d, f, h) Feature plots of gene expression levels in wild type and *gl* mutant, with gray indicating no expression and pink the highest expression. (c, e, g, i) Violin plots of gene expression levels in the indicated selected cell clusters, with wild type in red and *gl* in green. (b, c) *ato* expression is maintained further along the R8 trajectory in *gl* mutants. (d, e) The late photoreceptor marker *drl* shows strongly reduced expression in all photoreceptors in *gl* mutants. (f, g) The previously defined Gl target gene *pros* shows strongly reduced expression in R7 and cone cells in *gl* mutants. (h, i) The cone cell marker *NijC* shows strongly reduced expression in *gl* mutants.

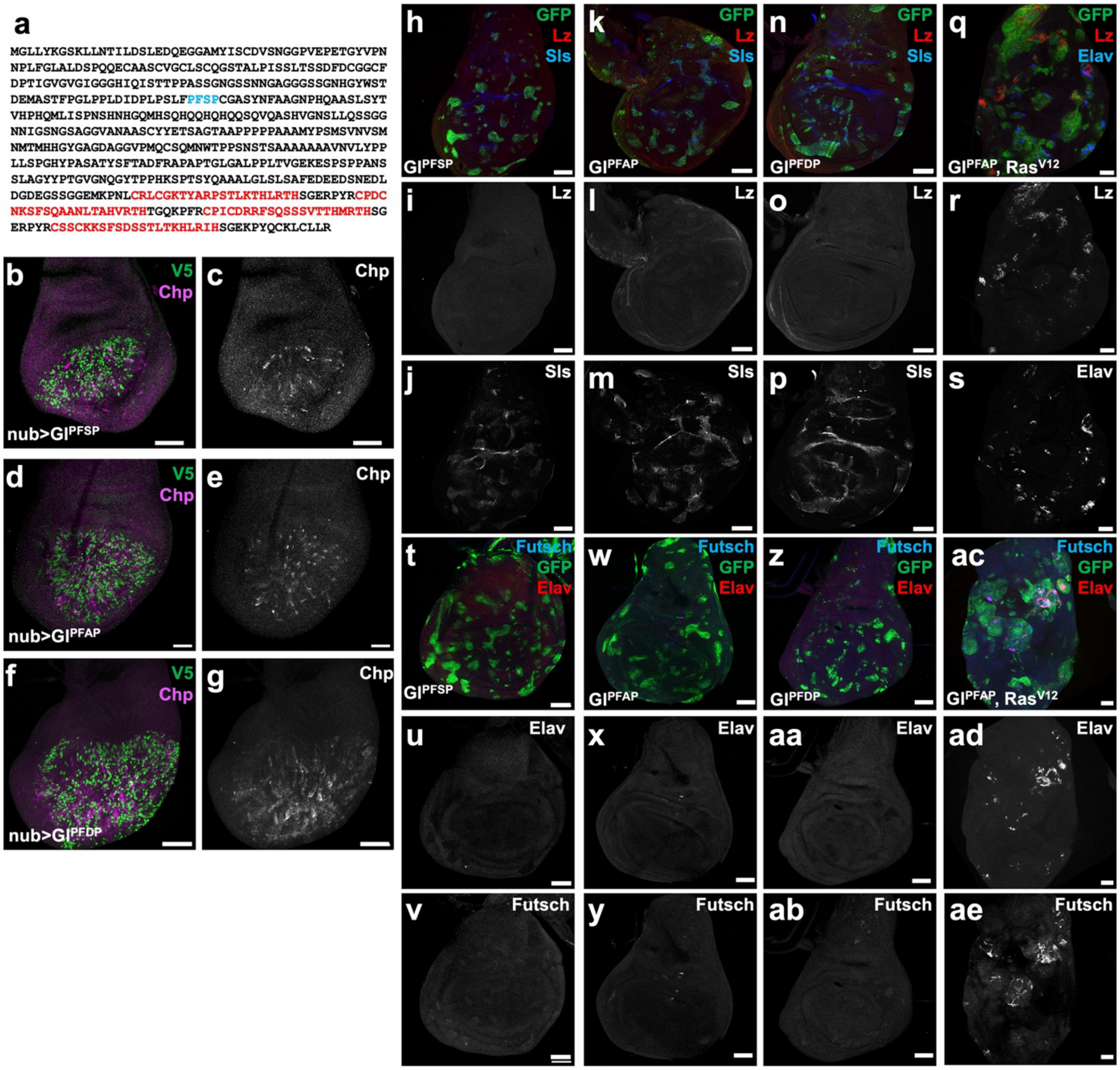

Supplementary Figure 2: A consensus MAPK site in Gl does not mediate synergy with EGFR signaling. (a) The Gl-RB amino acid sequence, showing the zinc fingers in red and the MAPK consensus motif in blue. (b-g) Third instar wing discs expressing wild-type *UAS-V5-Gl*^{PFSP} (b, c), non-phosphorylatable *UAS-V5-Gl*^{PFAP} (d, e) or phosphomimetic *UAS-V5-Gl*^{PFDP} (f, g) in the wing pouch with *nub-GAL4*, stained for V5 (green) and Chp (c, e, g, magenta in b, d, f). All constructs show similar expression levels and induce the Gl target gene Chp to a similar extent. n=13 (Gl^{PFAP}, Gl^{PFDP}), n=4 (Gl^{PFSP}). (h-ae) Third instar wing discs with clones labeled with GFP (green) that express *UAS-V5-Gl*^{PFSP} (h-j, t-v), U*AS-V5-Gl*^{PFAP} (k-m, w-y), *UAS-V5-Gl*^{PFDP} (n-p, z-ab)

or *UAS-V5-Gl*^{PFAP} and *UAS-Ras*^{V12} (q-s, ac-ae), stained for Lz (i, l, o, r, red in h, k, n, q), Sls (j, m, p, blue in h, k, n), Elav (s, u, x, aa, ad, blue in q, red in t, w, z, ac), or Futsch (v, y, ab, ae, blue in t, w, z, ac). n=4 (h-p), n=8 (q-s), n=6 (t-v, z-ab), n=10 (w-y), n=7 (ac-ae). Non-phosphorylatable Gl can still induce the cone cell marker Sls and synergize with Ras^{V12} to induce Lz, Elav and Futsch, and phosphomimetic Gl cannot induce Lz, Elav or Futsch. Scale bars, 50 μm.

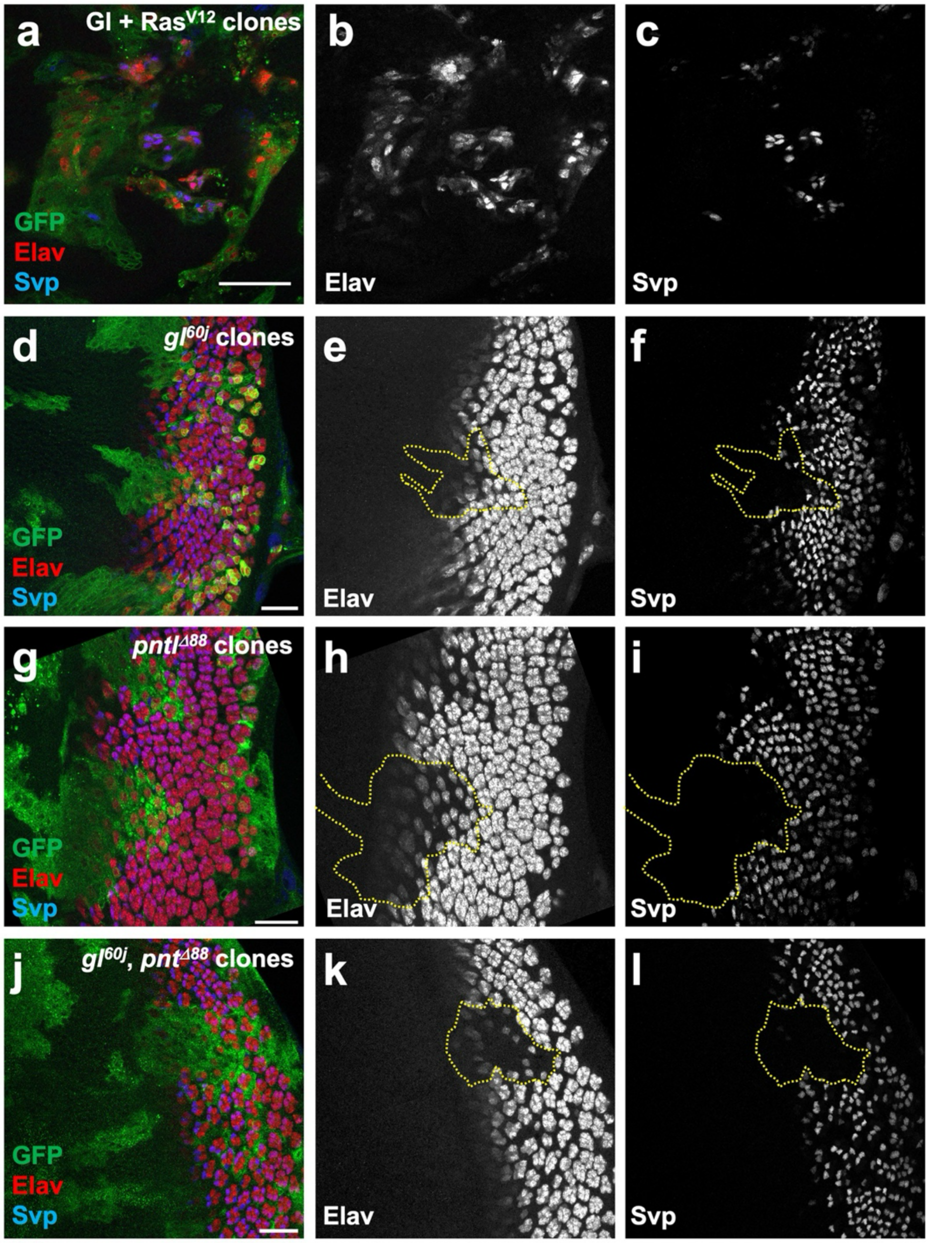

Supplementary Figure 3: *gl*, *pnt* double mutant clones resemble *pnt* single mutant clones. (a-c) show clones expressing UAS-Gl and UAS-Ras^{V12} in a third instar wing disc, labeled with GFP (green) and stained for Elav (b, red in a) and Seven-up (Svp), a marker for R1, R3, R4 and R6 (c, blue in a). Svp is expressed in a subset of the ectopic Elav-expressing cells. (d-l) show third instar eye discs with clones labeled with GFP (green) that are homozygous for gl^{60j} (d-f), $pnt^{\Delta 88}$ (g-i), or gl^{60j}, $pnt^{\Delta 88}$ (j-l), stained for Elav (e, h, k, red in d, g, j) and Svp (f, i, l, blue in d, g, j). Representative clones are

outlined in (e, f, h, I, k, l). n=8 (a-c), n=10 (d-f), n=11 (g-i), n=16 (j-l). Only single Elav-positive, Svp-negative photoreceptors differentiate in both *pnt* single mutant and *gl, pnt* double mutant clones, while *gl* single mutant clones include clusters with multiple Elav-positive photoreceptors, some of which express Svp. Scale bars, 50 μm (a-c), 20 μm (d-l).

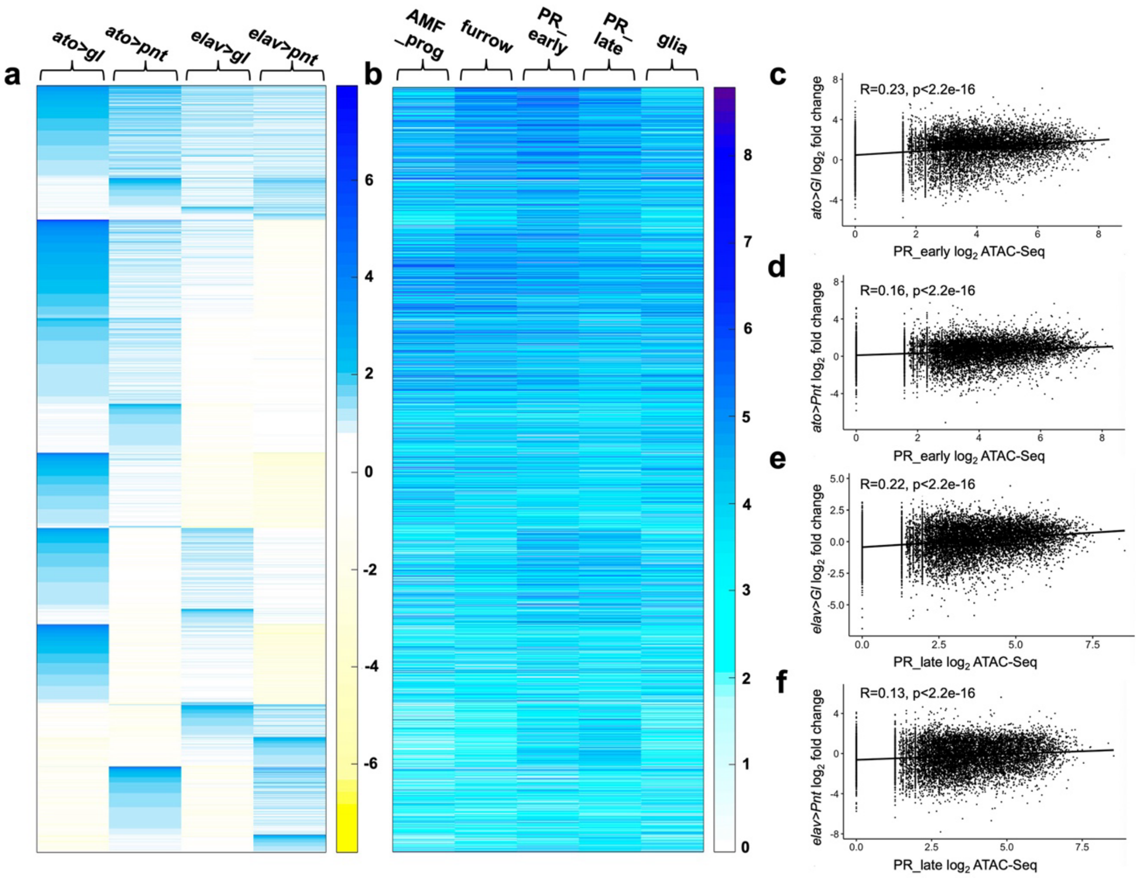

Supplementary Figure 4: ATAC-Seq chromatin accessibility level correlates with Gl and Pnt binding. Normalized ATAC-Seq pseudobulk counts were downloaded from the UCSC genome browser table browser http://genome.ucsc.edu/s/cbravo/Bravo_et_al_EyeAntennalDisc [41]. (a) DamID $\log_2$ fold changes were plotted in DamID peak regions that were significant in at least one condition, FDR<0.1, $\log_2$fc>1. Fold change is an indication of the size of the peak compared to the DamID control. K-means clustering was used to show patterns, k=8. (b) Average of normalized and $\log_2$ transformed ATAC-Seq counts in the same DamID peak regions plotted in A for direct comparison. (c) Correlation between normalized and $\log_2$ transformed ATAC-Seq counts in the peaks in early photoreceptor cells (b) and the DamID $\log_2$ fold changes in *ato>Gl* (a). (d) Correlation between early photoreceptor ATAC-Seq data and *ato>Pnt* DamID data. (e) Correlation between late photoreceptor ATAC-Seq data and *elav>Gl* DamID data. (f) Correlation between late photoreceptor ATAC-Seq data and *elav>Pnt* DamID data.

a

Gene symbol	Expression in photoreceptors	Mutant or RNAi line tested	Phenotype
scrt	yes	*scrt*^{jo11}	shown in Figure 6
scrape	yes	*scrape* ^{1E}, also double mutant with *scrt*	shown in Figure 6
nerfin-1	yes	P{TRiP.HMC04083}attP2	no phenotype
Sidpn	late	P{GD2559}v3065, P{GD14369}v27049	no phenotype
CG12071	late	P{TRiP.JF02332}attP2	no phenotype
CG14655	yes	P{TRiP.HMJ21158}attP40	no phenotype
CG43347	yes	P{TRiP.HMC04913}attP40	small rough eye
dati	low	P{TRiP.JF02251}attP2, P{KK104525}VIE-260B	no phenotype
onecut	yes	P{TRiP.HMJ22466}attP40	no phenotype
Drgx	very low	P{KK111117}VIE-260B, P{TRiP.HMC04912}attP40	rough eye for P{KK111117}VIE-260B, no phenotype for P{TRiP.HMC04912}attP40
Lim3	R8, R2, R5	*Lim3* ^{1}	no phenotype
Nfl	late	P{TRiP.JF02219}attP2	no phenotype
Pph13	late		late photoreceptor differentiation, already known
lz	R1, R6, R7		photoreceptor differentiation, already known
run	R7, R8		photoreceptor axon guidance, already known
oli	no		not tested
slbo	no		not tested
erm	no		not tested
dimm	no		not tested
CG15336	no		not tested

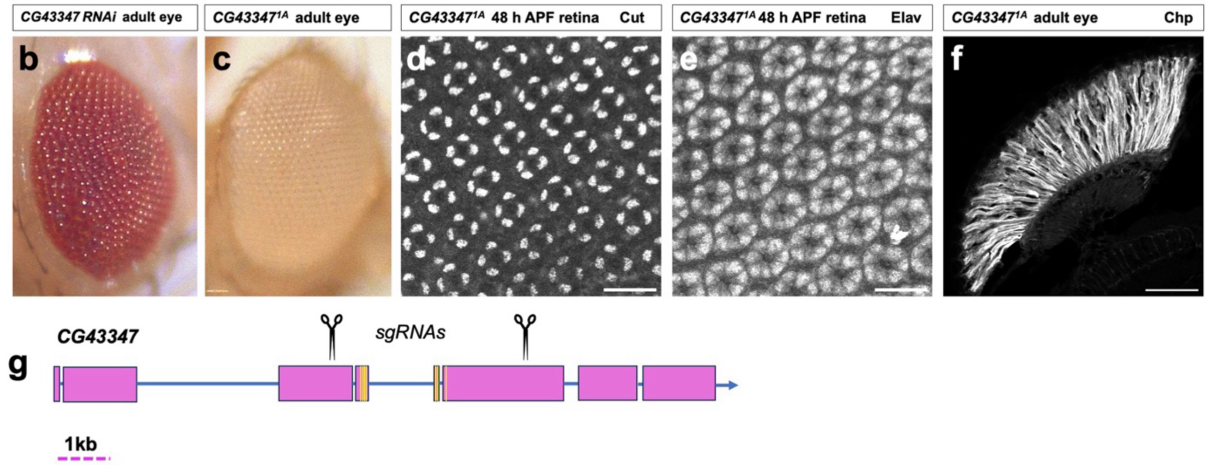

Supplementary Figure 5: Downstream transcription factors mediate only subsets of the Gl and Pnt functions. (a) chart indicating transcription factors that were synergistically induced by Gl and Ras^{V12} in a *pnt*-dependent manner in the wing disc. Expression in photoreceptors is based on our scRNA-Seq data. For those that were expressed in photoreceptors and had not been previously investigated in eye development (unshaded), the indicated mutants or RNAi lines were tested for an effect on the adult eye. RNAi lines were crossed to *ey3.5-FLP, Act>CD2>GAL4; UAS-dcr2*. As *CG43347* RNAi had a strong phenotype in this assay, deletion mutants were generated by CRISPR. (b) Adult eye in which *CG43347* RNAi was driven by *ey3.5-FLP, Act>CD2>GAL4* during eye development, showing roughness and reduced size. n=9. (c) Adult eye of *CG43347*^{1A} deletion mutant, which appears normal. (d, e) *CG43347*^{1A} homozygous mutant 48 h APF pupal retinas stained for Cut (d) or Elav (e), showing normal differentiation of cone cells and photoreceptors. n=6. (f) horizontal section of a

CG43347^{1A} homozygous mutant adult eye, stained for Chp. Photoreceptor rhabdomeres appear normal. n=3. Scale bars, 10 μm (d, e), 50 μm (f). (g) Schematic of the *CG43347* gene showing the two sgRNAs used. They are 3916 bp apart and span all the predicted zinc fingers (yellow).

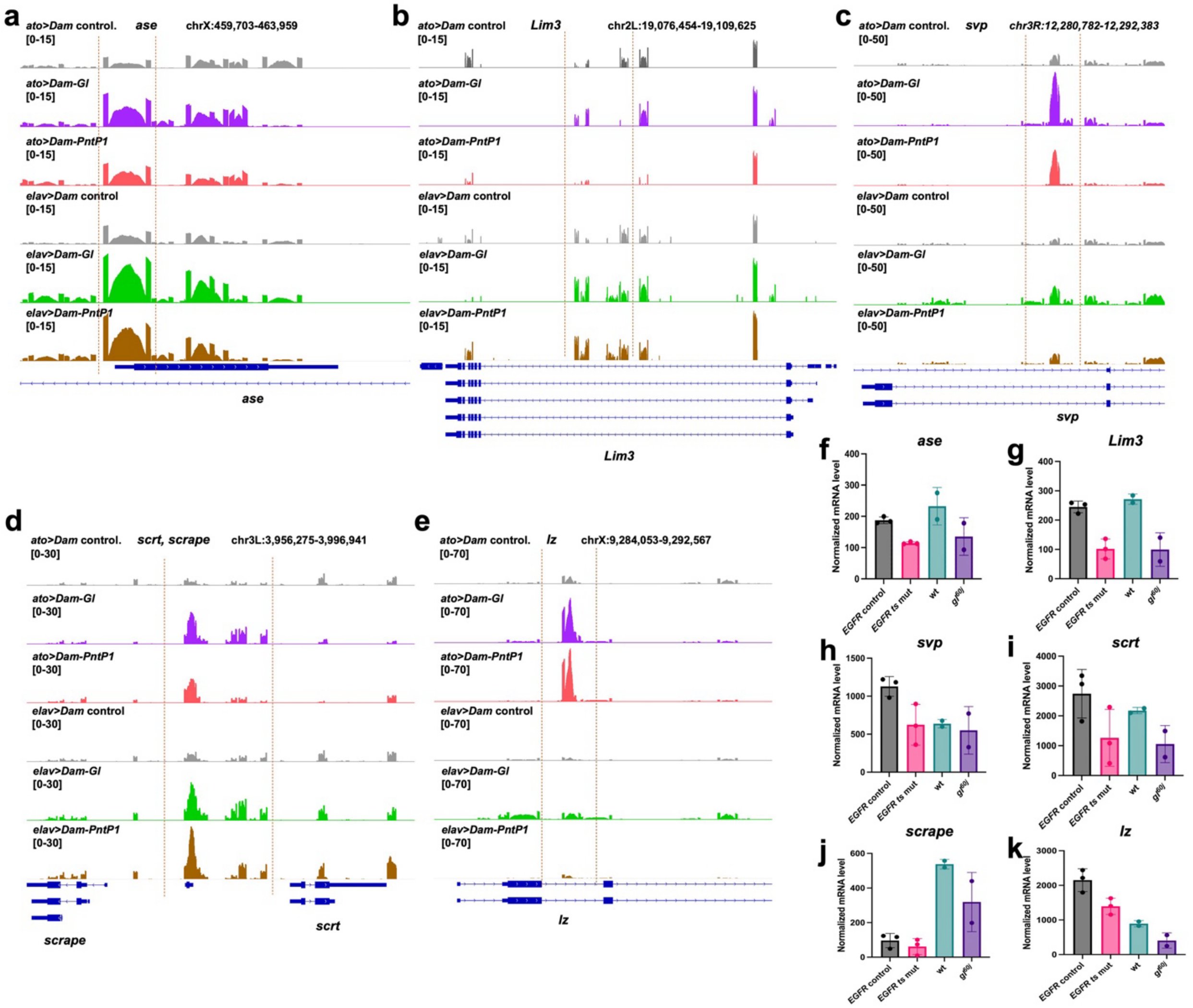

Supplementary Figure 6: DamID peaks and RNA-Seq data for the genes encoding intermediate transcription factors shown in Fig. 7. (a-e) Significant Dam-ID peaks are between the red dashed lines. (a) *ase*; (b) *Lim3*; (c) *svp*; (d) *scrt* and *scrape*; (e) *lz*. (f-k) Expression levels of these intermediate transcription factors from $EGFR^{ts}$ RNA-Seq (n=3 biological replicates) and *gl* RNA-Seq [35] analysis of larval eye discs.
