# Supplementary material for: Synergistic activation by Glass and Pointed promotes neuronal identity in the Drosophila eye disc
Source: Nat Commun. 2024 Aug 17;15:7091. doi: 10.1038/s41467-024-51429-z (PMC11330500; doi:10.1038/s41467-024-51429-z)
Supplement: Supplementary file 3 — Description of Additional Supplementary Files [file 41467_2024_51429_MOESM3_ESM.pdf]

Description of Additional Supplementary Files

File Name: Supplementary Data 1

Description: Marker genes for each cluster that are differentially expressed in *gl* mutants. For each cluster, a list of marker genes is given along with the average $\log_2$ fold change between wild-type and *gl* mutant eye discs, the proportion of cells in that cluster with detectable expression of the gene in wild type and in *gl*, and the adjusted p-value for the differential expression. Tab 1: R8; Tab 2: R2/R5; Tab 3: R3/R4; Tab 4: R1/R6; Tab 5: R7; Tab 6: cone cell cluster 6; Tab 7: cone cell cluster 11; Tab 8: cone cell cluster 21. p values were computed using FindMarkers in Seurat, based on the non-parametric Wilcoxon rank sum two-sided test. p values adjusted for multiple testing with the Bonferroni correction are also shown.

File Name: Supplementary Data 2

Description: PCA analysis of genes that vary in each cell type in wild-type and *gl* mutant eye discs. Tab 1: Summary of the numbers of genes with |PC1 loadings| > 0.01 in each photoreceptor cluster in *gl* but not wild-type, wild-type but not *gl*, or both. Tab 2: List of genes with |PC1 loadings| > 0.01 in *gl* but not wild-type in each photoreceptor cluster. Tab 3: List of genes with |PC1 loadings| > 0.01 in wild-type but not *gl* in each photoreceptor cluster. Tab 4: List of genes with |PC1 loadings| > 0.01 in both wild-type and *gl* in each photoreceptor cluster. Genes with |PC1 loadings| > 0.05 are highlighted in yellow in Tabs 2-4. Tab 5: Summary of the numbers of genes with |PC1 loadings| > 0.01 in each photoreceptor cluster in *gl* and/or wild-type that are among the *pnt*-dependent or *pnt*-independent genes induced by Gl and activated Ras in the wing disc, the *Egfr*-dependent genes found in RNA-Seq analysis of the eye disc, or the genes with Gl or Pnt DamID peaks in *ato-GAL4* and/or *elav-GAL4*-expressing cells. Tab 6: List of *pnt*-dependent genes induced by Gl and activated Ras in the wing disc that have |PC1 loadings| > 0.01 for each photoreceptor cluster and genotype. Tab 7: List of *pnt*-independent genes induced by Gl and activated Ras in the wing disc that have |PC1 loadings| > 0.01 for each photoreceptor cluster and genotype. Tab 8: List of *Egfr*-dependent genes in the eye disc that have |PC1 loadings| > 0.01 for each

photoreceptor cluster and genotype. Tab 9: List of genes with Gl DamID peaks in *ato-GAL4* and/or *elav-GAL4*-expressing cells that have |PC1 loadings| > 0.01 for each photoreceptor cluster and genotype. Tab 10: List of genes with Pnt DamID peaks in *ato-GAL4* and/or *elav-GAL4*-expressing cells that have |PC1 loadings| > 0.01 for each photoreceptor cluster and genotype.

File Name: Supplementary Data 3

Description: Gene expression levels in wing discs misexpressing Gl and activated Ras. Tab 1: RPKM values for gene expression in wing discs containing clones that express *UAS-GFP*, *UAS-GFP* and *UAS-Gl*, *UAS-GFP* and *UAS-Ras^{V12}*, *UAS-GFP*, *UAS-Gl* and *UAS-Ras^{V12}*, *UAS-GFP*, *UAS-Gl* and *UAS-Ras^{V12}* in *pnt* mutant clones, or *UAS-GFP* and *UAS-Gl* in *cic* mutant clones. All data are normalized to GFP expression except for *UAS-GFP* and *UAS-Ras^{V12}*, which is normalized to library size. Tab 2: List of synergistic genes that are *pnt*-dependent or *pnt*-independent. Synergy genes are defined as those for which overexpressing both Gl and Ras^{V12} increased the gene expression level significantly compared to overexpressing either Gl alone or Ras^{V12} alone. *pnt* dependency was defined as significantly lower gene expression in *pnt* mutant than wild-type wing discs that overexpressed both Gl and Ras^{V12}. Log$_2$ fold change>1 and FDR<0.1 for differential expression were the cut-off for significance. The normalization method was chosen as described in Tab 1. For the *pnt*-dependent synergistic genes, information about gene function and expression from Flybase is listed in column F. The 121 genes annotated as having neuronal functions or neuronal-specific expression are highlighted in blue. The clusters in which each gene is expressed in the eye disc in our wild-type scRNA-Seq analysis are listed in column G. Tab 3: log$_2$ of gene expression levels in the wing disc experiment for the 265 *pnt*-dependent synergistic genes. Tab 4: GFP expression levels of the three replicates of all genotypes, which were used as the basis for GFP normalization. Tab 5: Differential expression analysis comparing discs overexpressing Gl to discs overexpressing both Gl and Ras^{V12}. Tab 6: Differential expression analysis comparing discs overexpressing Ras^{V12} (library normalization) to discs overexpressing both Gl and Ras^{V12}. Tab 7: Differential expression analysis comparing discs overexpressing both Gl and Ras^{V12} in

wild-type clones to discs overexpressing the same genes in *pnt* mutant clones. Tab 8: Differential expression analysis comparing discs overexpressing Gl in wild-type clones to those overexpressing Gl in *cic* mutant clones. Tab 9: Full raw count table prior to any normalization. p values were computed using the two-tailed Wald test in DESeq2. p values adjusted for multiple testing by the Benjamini and Hochberg method are also included.

File Name: Supplementary Data 4

Description: Gene annotation and genomic coordinates of Dam-ID peaks bound by Gl and/or Pnt. Tab 1: Significant Gl and/or Pnt peaks compared to Dam control in *ato-GAL4* or *elav-GAL4* cells, FDR<0.1, log_2 fold change >1. Genomic coordinates, log_2 fold changes, FlyBase ID, p value and adjusted p value are indicated. p values were computed using the two-tailed Wald test in DESeq2. p values adjusted for multiple testing by the Benjamini and Hochberg method are also included. AG, *ato>gl*; AD, *ato>Dam*; AP, *ato>pnt*; EG, *elav>gl*; ED, *elav>Dam*; EP, *elav>pnt*. Tab 2: Genomic coordinates of significant peaks and log_2 fold change numbers for the data shown in Fig. 4a and Fig. S4a. Tab 3: FlyBase IDs of genes used in the UpSet plots in Fig. 4c-f. Tab 4: Gene symbols, genomic coordinates and log_2 fold change of peaks shown in Fig. 5c. Tab 5: ATAC-Seq pseudobulk normalized data used for Fig. 5d and e, including peak coordinates. Tab 6: Genomic coordinates, FlyBase IDs, and categorized genomic locations that were plotted in Fig. 5f; FlyBase IDs used for Fig. 5g; FlyBase IDs and gene symbols used for GO-term analysis in geneontology.org shown in Fig. 5h.

File Name: Supplementary Data 5

Description: Genes affected by EGFR inactivation in eye discs. RPKM values for gene expression in control $Egfr^{ts}/Egfr^{f2}$ eye discs maintained at the permissive temperature of 18°C (EGFR_WT) and in $Egfr^{ts}/Egfr^{f2}$ eye discs after 24 h at 29°C (EGFR_MUT). Tab 1: Significantly down-regulated genes in the $Egfr^{ts}/Egfr^{f2}$ mutant compared to control, cutoff is log_2 fold change >0.25, p<0.05, SD/mean<0.5. Tab 2: RPKM normalized gene expression data without differential analysis. Tab 3: the same table with functional annotation of selected genes of interest. p values were computed

using the two-tailed Wald test in DESeq2. p values adjusted for multiple testing by the Benjamini and Hochberg method are also included.
