# Supplementary material for: Synergistic activation by Glass and Pointed promotes neuronal identity in the Drosophila eye disc
Source: Nat Commun. 2024 Aug 17;15:7091. doi: 10.1038/s41467-024-51429-z (PMC11330500; doi:10.1038/s41467-024-51429-z)
Supplement: Supplementary file 9 — Reporting Summary [file 41467_2024_51429_MOESM9_ESM.pdf]

nature portfolio

Corresponding author(s): Jessica Treisman

Last updated by author(s): Jul 17, 2024

Reporting Summary

Nature Portfolio wishes to improve the reproducibility of the work that we publish. This form provides structure for consistency and transparency in reporting. For further information on Nature Portfolio policies, see our Editorial Policies and the Editorial Policy Checklist.

Statistics

For all statistical analyses, confirm that the following items are present in the figure legend, table legend, main text, or Methods section.

n/a	Confirmed	
☐	☒	The exact sample size (*n*) for each experimental group/condition, given as a discrete number and unit of measurement
☐	☒	A statement on whether measurements were taken from distinct samples or whether the same sample was measured repeatedly
☐	☒	The statistical test(s) used AND whether they are one- or two-sided<br>*Only common tests should be described solely by name; describe more complex techniques in the Methods section.*
☒	☐	A description of all covariates tested
☐	☒	A description of any assumptions or corrections, such as tests of normality and adjustment for multiple comparisons
☐	☒	A full description of the statistical parameters including central tendency (e.g. means) or other basic estimates (e.g. regression coefficient) AND variation (e.g. standard deviation) or associated estimates of uncertainty (e.g. confidence intervals)
☐	☒	For null hypothesis testing, the test statistic (e.g. *F*, *t*, *r*) with confidence intervals, effect sizes, degrees of freedom and *P* value noted<br>*Give P values as exact values whenever suitable.*
☒	☐	For Bayesian analysis, information on the choice of priors and Markov chain Monte Carlo settings
☒	☐	For hierarchical and complex designs, identification of the appropriate level for tests and full reporting of outcomes
☒	☐	Estimates of effect sizes (e.g. Cohen's *d*, Pearson's *r*), indicating how they were calculated

Our web collection on statistics for biologists contains articles on many of the points above.

Software and code

Policy information about availability of computer code

Data collection	For scRNA-Seq, data was collected using the Cell Ranger v6.0.1 count pipeline and the Drosophila melanogaster reference genome Release 6 (Dm6). Bulk RNA-Seq data were mapped to the genome with STAR aligner (v2.5.0c), Picard tools (v.1.126), HTSeq (v0.6.0) and DESeq2. Bigwig files were generated with BEDTools (v2.17.0) and bedGraphToBigWig tool (v4). DamID data were generated with Bowtie2 (v2.2.4), Picard tools (v.1.126), BEDTools (v.2.17.0), bedGraphToBigWig tool (v.4), MACS (v1.4.2), DESEq2 and ChIPseeker (v1.8.0).
Data analysis	scRNA-Seq data were analyzed using Seurat v4.2.1 including Seurat SCTransform, Seurat RunPCA , UMAP, FindNeighbors, FindClusters, scCustomize, Function FeaturePlot_scCustom, SplitObject and SeuratWrappers. Monocle 3 was used for trajectory analysis. Bulk RNA-Seq data and DamID data were analyzed in R environment (v3.1.1) including the EnhancedVolcano and UpSetR packages and using Matlab (R2022b), IGV: Integrative Genomics Viewer and MEME-Suite STREME. Specific code used is available at https://github.com/hw1804/JTreis_Gl_Ras_project. Images were analyzed using ImageJ/Fiji.

For manuscripts utilizing custom algorithms or software that are central to the research but not yet described in published literature, software must be made available to editors and reviewers. We strongly encourage code deposition in a community repository (e.g. GitHub). See the Nature Portfolio guidelines for submitting code & software for further information.

Data

Policy information about availability of data

All manuscripts must include a data availability statement. This statement should provide the following information, where applicable:

- Accession codes, unique identifiers, or web links for publicly available datasets
- A description of any restrictions on data availability
- For clinical datasets or third party data, please ensure that the statement adheres to our policy

All the raw data for scRNA-seq, bulk RNA-Seq and DamID-Seq have been archived online with Gene Expression Omnibus (GEO) with the accession number GSE256221 (https://www.ncbi.nlm.nih.gov/geo/query/acc.cgi?acc=GSE256221). Source data are provided with this paper.

Research involving human participants, their data, or biological material

Policy information about studies with human participants or human data. See also policy information about sex, gender (identity/presentation), and sexual orientation and race, ethnicity and racism.

Reporting on sex and gender	*Use the terms sex (biological attribute) and gender (shaped by social and cultural circumstances) carefully in order to avoid confusing both terms. Indicate if findings apply to only one sex or gender; describe whether sex and gender were considered in study design; whether sex and/or gender was determined based on self-reporting or assigned and methods used.*<br>*Provide in the source data disaggregated sex and gender data, where this information has been collected, and if consent has been obtained for sharing of individual-level data; provide overall numbers in this Reporting Summary. Please state if this information has not been collected.*<br>*Report sex- and gender-based analyses where performed, justify reasons for lack of sex- and gender-based analysis.*
Reporting on race, ethnicity, or other socially relevant groupings	*Please specify the socially constructed or socially relevant categorization variable(s) used in your manuscript and explain why they were used. Please note that such variables should not be used as proxies for other socially constructed/relevant variables (for example, race or ethnicity should not be used as a proxy for socioeconomic status).*<br>*Provide clear definitions of the relevant terms used, how they were provided (by the participants/respondents, the researchers, or third parties), and the method(s) used to classify people into the different categories (e.g. self-report, census or administrative data, social media data, etc.)*<br>*Please provide details about how you controlled for confounding variables in your analyses.*
Population characteristics	*Describe the covariate-relevant population characteristics of the human research participants (e.g. age, genotypic information, past and current diagnosis and treatment categories). If you filled out the behavioural & social sciences study design questions and have nothing to add here, write "See above."*
Recruitment	*Describe how participants were recruited. Outline any potential self-selection bias or other biases that may be present and how these are likely to impact results.*
Ethics oversight	*Identify the organization(s) that approved the study protocol.*

Note that full information on the approval of the study protocol must also be provided in the manuscript.

Field-specific reporting

Please select the one below that is the best fit for your research. If you are not sure, read the appropriate sections before making your selection.

[x] Life sciences   [ ] Behavioural & social sciences   [ ] Ecological, evolutionary & environmental sciences

For a reference copy of the document with all sections, see nature.com/documents/nr-reporting-summary-flat.pdf

Life sciences study design

All studies must disclose on these points even when the disclosure is negative.

Sample size	Three biological replicates were performed for RNA-Seq and DamID experiments, as this is the standard in the field. Sample sizes for phenotypic analysis were not predetermined by statistical methods, but were chosen based on prior experience to be sufficient to observe statistically significant differences for the relatively large effect sizes we observed.
Data exclusions	No data were excluded from the analysis.
Replication	Three replicates were performed for all genomics experiments, and at least three for all phenotypic analysis experiments. All attempts at replication were successful.
Randomization	Samples were assigned based on genotype, so randomization was not appropriate.
Blinding	Bioinformatics analysis was done by individuals unfamiliar with the expected results for each genotype. Phenotypic analysis was not carried out blind, as the differences between genotypes were large enough to prevent effective blinding.

Reporting for specific materials, systems and methods

We require information from authors about some types of materials, experimental systems and methods used in many studies. Here, indicate whether each material, system or method listed is relevant to your study. If you are not sure if a list item applies to your research, read the appropriate section before selecting a response.

Materials & experimental systems

n/a	Involved in the study
☐	☒ Antibodies
☒	☐ Eukaryotic cell lines
☒	☐ Palaeontology and archaeology
☐	☒ Animals and other organisms
☒	☐ Clinical data
☒	☐ Dual use research of concern
☒	☐ Plants

Methods

n/a	Involved in the study
☒	☐ ChIP-seq
☒	☐ Flow cytometry
☒	☐ MRI-based neuroimaging

Antibodies

Antibodies used	Chicken anti-GFP (1:200, Invitrogen A10262), rat anti-Elav (1:50; Developmental Studies Hybridoma Bank (DSHB) Rat-Elav-7E8A10), mouse anti-Futsch (1:20, DSHB 22C10), mouse anti-Pros (1:10, DSHB Prospero MR1A), rabbit anti-dsRed (1:400, TaKaRa Living Colors® Polyclonal 632496), and mouse anti-Chp (1:25; DSHB 24B10).
Validation	Rat anti-Elav (https://dshb.biology.uiowa.edu/Rat-Elav-7E8A10-anti-elav), mouse anti-Futsch (https://dshb.biology.uiowa.edu/22C10), mouse anti-Pros (https://dshb.biology.uiowa.edu/Prospero-MR1A) and mouse anti-Chp (https://dshb.biology.uiowa.edu/24B10) have been used extensively in our lab and many others. Numerous references are cited on their Developental Studies Hybridoma Bank web pages. Chicken anti-GFP has 374 references on the ThermoFisher website (https://www.thermofisher.com/antibody/product/GFP-Antibody-Polyclonal/A10262). Rabbit anti-dsRed has been validated in the Drosophila brain (Nicolai et al. 2010, PNAS 107 (47) 20553-20558, https://doi.org/10.1073/pnas.1010198107).

Animals and other research organisms

Policy information about studies involving animals; ARRIVE guidelines recommended for reporting animal research, and Sex and Gender in Research

Laboratory animals	Drosophila melanogaster mutants and transgenic lines were studied at third instar larval, mid-pupal and adult stages. Strains used were UAS-GFP; tub-GAL4, FRT82, tub-GAL80/TM6B (https://pubmed.ncbi.nlm.nih.gov/29324767/), UAS glRB; FRT82, UAS-RasV12, pntΔ88/SM6-TM6B (generated from FBal0346371, FBal0060587, FBal0035437, FBti0002074), UAS-glRB; FRT82, cicQ219X/SM6-(TM6B (generated using FBal0220444), FRT82, UAS-RasV12 (generated from FBal0060587), UAS-glRB; FRT82 (generated from FBal0346371), UAS-glRB; FRT82, UAS-RasV12/SM6-TM6B (generated from FBal0060587, FBal0346371), ato3'FL-GAL4 (https://pubmed.ncbi.nlm.nih.gov/25980363/), elav-GAL4/SM6-TM6B (FBal0042579), UAS-Dam (https://pubmed.ncbi.nlm.nih.gov/27490632/), Egfrts1a (FBal0083481), Egfrf2 (FBal0003530), ey3.5-FLP, Act>CD2>GAL4; UAS-dcr2 (generated from FBti0141243, FBtp0001640, FBal0211026), attP40 (FBti0114379), nos-Cas9 ZH-2A (FBti0159183), FRT80, scrtj011/TM6B (FBal0046413), ey-FLP, gl-lacZ; lGMR-GAL4, UAS-myrTomato/CyO; FRT80, tub-GAL80/TM6B (generated using FBti0015982, FBti0015985, FBti0058798, FBti0131969, FBti0002073, FBti0012693), w1118 (FBal0018186), nos-Cas9/CyO (FBti0199256), attP2 (FBti0040535), gl60j (https://pubmed.ncbi.nlm.nih.gov/2770860/)
Wild animals	N/A
Reporting on sex	Only males were used for the scRNA-Seq experiment in order to sample genes on the Y chromosome. Both males and females were used interchangeably for other experiments, as no sex-specific differences in eye development have been observed.
Field-collected samples	N/A
Ethics oversight	No ethical approvals were required, as the study used only invertebrates (Drosophila).

Note that full information on the approval of the study protocol must also be provided in the manuscript.

Plants

Seed stocks	N/A
Novel plant genotypes	N/A
Authentication	N/A
